# A rare ovarian hilus cell tumour accompanying bilateral serous cystadenomas: report of a case

**DOI:** 10.1186/s13048-021-00833-w

**Published:** 2021-06-21

**Authors:** Haider Ali Malakzai, Jamshid Abdul-Ghafar, Abdul Sami Ibrahimkhil, Ahmed Maseh Haidary

**Affiliations:** Department of Pathology and Clinical Laboratory, French Medical Institute for Mothers and Children (FMIC), Kabul, Afghanistan

**Keywords:** Hilus cell tumour, Steroid cell tumour, Ovarian tumour, Sex cord-stromal tumours, Serous cystadenomas

## Abstract

**Background:**

Hilus cell tumours is considered an uncommon branch of androgen producing neoplasms that accounts for < 5% of all ovarian tumours. They are mostly benign and have characteristic gross and microscopic features. Here we present the first case of a hilus cell tumour in association with bilateral serous cystadenomas.

**Case presentation:**

A 65-year-old lady with no symptoms of virilization, presented with postmenopausal dysfunctional uterine bleeding and radiological investigations revealing bilateral ovarian cysts that required a total abdominal hysterectomy with bilateral salpingo-oophorectomy. Gross and microscopic evaluation confirmed the diagnosis of hilus cell tumour associated with bilateral serous cystadenomas.

**Conclusions:**

This was the first case of hilus cell tumour in association with bilateral serous cystadenomas of the ovaries. Although, majority of hilus cell tumours that have been reported in the literature were benign, further studies are required to determine the behavior of the disease.

## Background

Hilus cells of the ovary are ultrastructurally similar to Leydig cells of the testis except for the difference in chromatin pattern [1, 2]. Berger was the first person to describe the morphology of these cells in 1922, and he referred to them as “sympathicotropic” cells [2].

Hilus Cell Tumours (HCT) is a rare branch of androgen producing neoplasms that account for < 5% of all ovarian tumours [3]. Most of the hilus cell tumours documented are small in size and benign in nature, except for two cases, of which the first one was described by Stewart RS and Woodard DE, in the left side broad ligament of a 67-year-old lady resulting in local extension with distant metastasis [4], and the second case was reported by Charles RE and Harry EH, reporting a metastasizing HCT in a 60-year-old female [5].

The morphological and immunohistochemical (IHC) profile of HCTs are identical to others Steroid Cell Tumours (SCT) of the ovary including; steroid cell tumours - not otherwise specified, recurrent steroid cell tumours and stromal luteoma. However, the presence of cytoplasmic inclusions known as “Reinke Crystals (RC)” is pathognomonic for HCT, despite their appearance in about half of the cases [1, 6, 7]. Here we present the first case of HCT accompanying bilateral Serous Cystadenomas (SC).

## Case presentation

A 65-year-old Afghan lady with no symptoms of virilization, presented with history of postmenopausal dysfunctional uterine bleeding. Radiological investigations showed bilateral ovarian cysts and the patient was elected for surgery to undergo a total abdominal hysterectomy with bilateral salpingo-oophorectomy. We received the formalin fixed surgical specimen coded as “uterus with ovaries”.

**Fig. 1:**
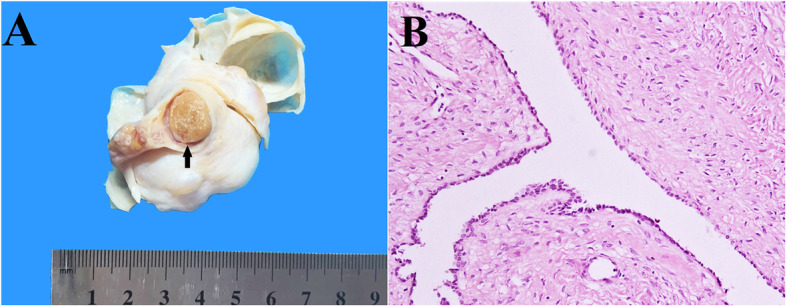
**A**, **B**. Gross and high-power microscopic presentation of right ovary. Grossly, the ovary shows a gray-white multilocular cyst, along with a well-circumscribed HCT, that reveals gray-yellow gelatinous appearance (**A**, arrow). The microscopic examination of ovarian cyst reveals serous cystadenoma characterized by fibrocollagenous cyst-wall fragments showing simple cuboidal epithelial cell lining (**B**)

**Gross examination** of the specimen revealed a uterus with cervix and attached bilateral adnexae. Upon serial sectioning of the uterus, the myometrium showed multiple small hemorrhagic foci. Ovaries were enlarged in size, cystically dilated with gray-white smooth external surfaces (Fig. 1A). Upon opening, the ovarian cysts were multilocular, filled with yellowish watery fluid and had smooth inner surfaces. In addition, there was a solid well-circumscribed mass of approximately 1.4 cm in its greatest diameter, located at the hilum of the right ovary (Fig. 1A). The mass was oval in shape with smooth external surface and upon dissection, it exhibited gray-yellow gelatinous cut surface (Fig. 1A). The cervix and both the attached fallopian tubes were grossly unremarkable.

**Microscopic examination** of the Hematoxylin and Eosin (H&E) stained sections of myometrium revealed foci of adenomyosis characterized by the presence of benign endometrial glands with stroma inside myometrial layer. The sections of bilateral ovarian cysts revealed cyst-wall fragments composed of fibrocollagenous tissue, lined by single layer of benign cuboidal cells with centrally placed bland nuclei (Fig. 1B). No evidence of atypia, dysplastic changes or increased mitotic figures was seen and on the basis of these findings, the diagnosis of serous cystadenoma was made.

**Fig. 2:**
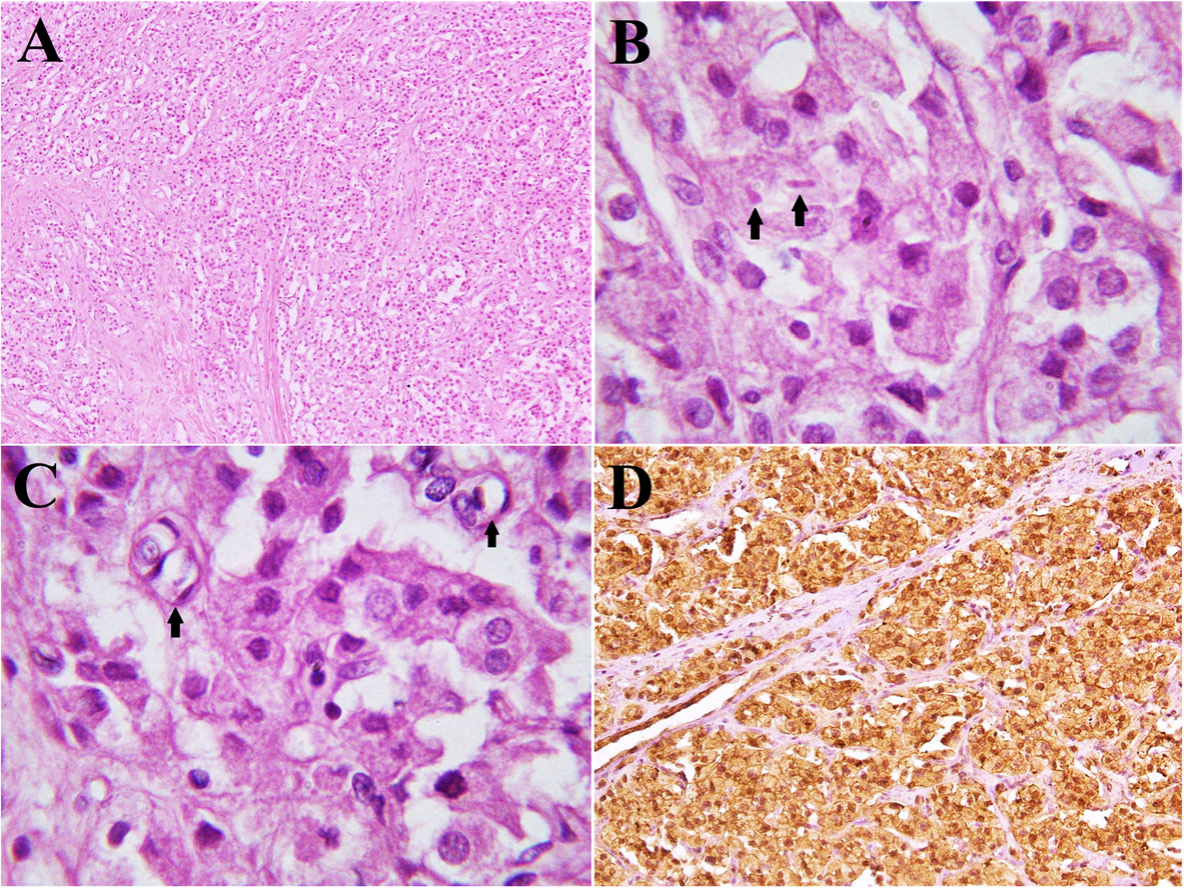
**A**, **B**, **C**, **D**. Intermediate and high-power microscopic examination of HCT. The tumour is arranged in sheets and lobules of neoplastic polygonal cells separated by fibrous bands (**A**). High-power examination of tumour reveals the presence of RC in the cytoplasm of occasional cells (**B**, arrows), along with lipid-rich cells, which show large vacuoles of lipids in the cytoplasm (**C**, arrow). The tumour cells show diffuse and strong nuclear and cytoplasmic expression for calretinin (**D**)

The sections of the right ovarian hilar mass, composed of neoplastic cells, arranged in sheets and lobules, separated by fibrous bands (Fig. 2A). The individual neoplastic cells were large in size, polygonal in shape having distinct cell borders, abundant acidophilic granular cytoplasm, round vesicular nuclei and coarse chromatin (Fig. 2A). Sections of the tumour showed rare mitotic figures but no necrosis. Occasional rod-shaped cytoplasmic inclusions known as RC were seen (Fig. 2B) along with lipid-rich cells, in which lipids were present in large cytoplasmic vacuoles (Fig. 2C). IHC stain for calretinin (monoclonal mouse antibody; clone DAK-Calret 1, Dako Denmark A/S) was performed on formalin-fixed, paraffin-embedded tissue. After deparaffinization of the section, antigen retrieval included; placing PT module (Thermo Scientific, USA) in Tris/EDTA buffer (pH 9) (EnVision FLEX Target Retrieval Solution, 50x concentrated, Denmark) at 97 °C for 20 min and then cooling for 20 min at room temperature. Peroxidase blocking reagent was applied for 5 min to block exogenous peroxidase activity. The slides were then washed, afterward, primary antibody incubation was done for 20–25 min at room temperature. Subsequently, it was washed with tris-buffered saline and was incubated with dextran-coupled with peroxidase molecules (EnVision FLEX/HRP, Denmark) and secondary antibody for 20–25 min in an incubator, then washed in wash buffer and incubated with chromogen solution (EnVision FLEX DAB+Chromogen, Denmark) for 45 s to 1 min and washed again in wash buffer, and counterstained with Hematoxylin (Merck KGaA, Germany). Finally coverslipped for microscopic evaluation which revealed strong nuclear and cytoplasmic expressions in neoplastic cells, confirming the diagnosis of HCT (Fig. 2D). In addition to that, normal mesothelial cells and tissue from myometrium were used as positive and negative controls for calretinin respectively. Sections of the uterine cervix and both fallopian tubes showed no significant pathological changes.

## Discussion

In the recent World Health Organization (WHO) classification of tumours of female reproductive organs, HCT were classified as a separate entity in the category of Sex Cord-Stromal Tumours (SCST)– pure stromal tumours [7]. Most of these tumours originates from the hilum of the ovary, but a small number of them can be found in the ovarian stroma with the latest known as non-hilar type [7, 8]. There are three principal features that differentiate the tumours located at the hilum of the ovary from other ovarian SCTs, which are: the usual small size, occurrence in elderly women and having excellent prognosis [9]. Almost all the HCTs are unilateral with only a few reported cases showing bilateral existence [7, 10]. Most of the HCTs are small in size with the mean diameter of 2.4 cm and solid red-brown to yellow cut surface [7]. The majority of the HCT cells are polygonal or oval in shape with occasional elongated forms and the cell size ranging between 14 and 25 μm [2]. Predominantly, these cells have basophilic oval nuclei with coarse chromatin and abundant acidophilic granular cytoplasm [2].

The RC are pathognomonic for HCT, despite their appearance in about half of the cases [1, 6]. The RC are acidophilic rod-like cytoplasmic inclusions, often equal to the greatest diameter of the cell, which are generally seen in the Leydig cells of the testis but are also found in the hilus cells of the ovary [2]. Other cytoplasmic inclusions, which can be found in HCT, but are not specific, include; cytoplasmic vacuoles of lipids and small round granules of golden-brown pigments [2]. Although the IHC profiles of SCST are somehow similar, the studies suggest that particular staining patterns for calretinin may help to differentiate between the four main types of SCST; where HCT and the Leydig cell component of Sertoli-Leydig cell tumours show diffuse and strong nuclear and cytoplasmic expression, fibrothecomas lack such expressions [11].

HCTs are androgen secreting tumours and due to this feature, patients often present with the symptoms of virilization [12]. These tumours have been reported in association with other female genital tract’s benign and malignant neoplasms including; uterine myoma, polycystic ovaries, endometrial adenocarcinoma and granulosa cell tumour of the ovary [12–15]. It is believed that the ovarian hilum provides a niche for putative cancer-prone stem cells which show increased transformation potential after inactivation of some specific tumour suppressor genes and are suggested to be tumour-initiating [16, 17]. The latest studies reveal that cancer stem cells of the ovary not only account for tumour progression, but building out chemoresistance [18].

Considering the tumour size and the number of mitotic figures, most of the patients are deemed to have low grade HCT and thus require no further interventions. Still, there is need for further clinicopathological and molecular research to identify markers of prognostic significance [19].

## Conclusion

To the best of our knowledge, this was the first case of hilus cell tumour in association with bilateral serous cystadenomas of the ovaries. Available literature regarding HCT is scarce and considering the fact that majority of the so far reported cases were benign in their behavior, extensive scrutiny in to the ethiopathogenesis of the tumour is required to elaborate further upon the natural history of the tumour.

## Data Availability

All the generated data are included in this article.

## Abbreviations

HCT: Hilus cell tumour
SCT: Steroid cell tumour;
RC: Reinke crystals
H&E: Hematoxylin and eosin
SCST: Sex cord-stromal tumour
WHO: World Health Organization.
